# Mammary gland, skin and soft tissue tumors in pet cats: findings of the feline tumors collected from 2002 to 2022

**DOI:** 10.3389/fvets.2024.1320696

**Published:** 2024-08-14

**Authors:** Roberta Giugliano, Filippo Dell'Anno, Livia De Paolis, Maria Ines Crescio, Valentina Ciccotelli, Barbara Vivaldi, Elisabetta Razzuoli

**Affiliations:** ^1^National Reference Center of Veterinary and Comparative Oncology (CEROVEC), Istituto Zooprofilattico Sperimentale del Piemonte, Liguria e Valle d'Aosta, Genoa, Italy; ^2^Department of Medical Sciences, University of Turin, Turin, Italy; ^3^Department of Public Health, Experimental and Forensic Medicine, Section of Biostatistics and Clinical Epidemiology, University of Pavia, Pavia, Italy

**Keywords:** animals tumors registry, cadmium pollution, feline oncology, pet epidemiology, proportional morbility ratio (PMR)

## Abstract

**Introduction:**

Cancer is a leading cause of death in cats, and the rate of such disease has been increasing recently. Nonetheless, feline oncology represents an important area of study not only for the health and wellbeing of cats but also for human health since various types of cancer in cats share similarities to those found in humans. Therefore, epidemiological studies on feline oncology may suggest environmental and genetic factors contributing to cancer in cats, which can eventually be translated to improve human cancer care.

**Method:**

To provide an initial understanding of the epidemiology of feline neoplasms, a descriptive study was undertaken using a dataset documenting cases of feline cancer gathered from the Liguria region (northwest Italy) spanning from 2002 to 2022. The database includes tumor location, morphological codes of the International Classification of Diseases for Oncology, 3rd Edition (ICD-O-3), feline's breed, sex, neuter status, date of birth, date of diagnosis, national territorial unit code of the town of the owner's residence, and an alphanumeric string uniquely identifying the owner's surname.

**Results and discussion:**

The dataset involves a population of 4,399 cats, including 3,195 females (1,425 neutered) and 1,204 males (750 neutered). Our results indicate that mammary gland tumors are the most represented tumors in the female population, while soft tissue and skin cancers appear to have a higher abundance in the male population during the periods investigated (2002–2022). Moreover, Poisson regression analysis showed that not neutered female cats have a significantly increased risk of developing mammary gland tumors compared to the neutered female population [proportional morbidity ratio (PMR) neutered vs. not neutered = 0.58, 95% CI: 0.47–0.72]; meanwhile, for both sexes, for soft tissue and skin tumors, being neutered appears to be a risk factor (PMR neutered vs. not neutered = 2.26, 95% CI: 1.86–2.73; PMR neutered vs. not neutered = 1.16, 95% CI: 0.89–1.51). Finally, the evaluation of the Ligurian municipalities pollution, based on wild boars data (i.e., biomonitors), which coexisted with cats, was correlated to cancer development for all the tumors investigated (in polluted areas, estimated PMRs ranged from 42.61 to 80.13, 95% CI: 29.94–105.11). Overall, the data presented here suggest the use of the feline population as a possible animal model for human health, i.e., sentinel.

## 1 Introduction

Animal tumor registries (ATRs) are few and scattered (1–3). While it is true that human cancer registries have been regulated by law (3), Veterinary Medicine Cancer Registries have been discontinued and characterized, in many cases, by a poor organization (1–3). Until recently, cases were often recorded manually, with samples accompanied by paper case formats. Web-based advent and mandatory fields facilitated a more complete data collection (4). The first companion animal cancer registries started in the early 1960s in the USA with the Kansas University Neoplasm Registry (5–8) and the California Animal Neoplasm Registry (9–11), and, at that time, felines were not initially considered. A feline and canine registry was founded in Tulsa in 1972 (12), but it stopped operations 5 years later (13). In 2019, the University of Queensland established the first Australian registry, the ACARCinom network, enabling access to datasets suitable for identifying animal patterns and trends using retrospective data obtained from the Veterinary Laboratory Services (13). In 2020, the Vet-OncoNet platform, a Portuguese project inspired by the One Health vision, was launched. Recently, in the USA, the Veterinary Oncology Market Committee from the Veterinary Cancer Society (VCS) started collaborating with national laboratories to establish the incidence of neoplasms in pet animals (13). In the United Kingdom, the University of Liverpool runs the Small Animal Veterinary Surveillance Network (SAVSNET), a pathology-based Animal Tumor Registry (14). A large contribution to animal registries was provided by the Global Initiative for Veterinary Cancer Surveillance (GIVCS) (13, 15), helping to standardize and guide current and future veterinary cancer registries to determine the global loads of animal cancer and to identify and track changes in cancer burden between populations and species over time. In Italy, ATRs are present in Genoa (from 1985) (16), Venice and Vicenza (from 2005) (17), Lazio (from 2009) (18), Campania (from 2012 with the L.R. n. 19/12) (19), Umbria (from 2014) (20), and Marche regions (from 2015) (21). However, the extension to the whole Italian territory is far from complete. The Italian Network of Laboratories for Veterinary Oncology (NILOV) (22) was created in 2013 to collect diagnoses of pet tumors from multiple sources into a single database and facilitate collaboration. The creation and strengthening of ATR are crucial since animals, especially pets, could be sentinels for human health risks (23). Pets share outdoor and indoor environments with humans, thereby being exposed to the same environmental pollution. Due to the differences in body weight and metabolism between humans and animals, it may be more susceptible than humans to hazardous compounds (4, 13, 14, 23). Moreover, cancer in pets and humans shares similar histological features, genetic alterations, biological behavior, and cancer biology. Additionally, a pet's shorter life provides a quicker pathology occurrence and diagnosis (2, 24). These common points lay the foundation of comparative oncology.

For many years, the study of cancer cell lines has been conducted with an elective experimental model, including syngeneic or immunodeficient mice, humanized mice, and genetically engineered mice (GEM) that spontaneously develop tumors (25, 26). However, the human and mouse immune systems show discrepancies, and the murine model has been overcome by human primates (NHPs) and pet animals (25). Comparative studies in dogs are the most widespread as more data are available due to mandatory dog microchipping, which is very common throughout the European Union (27, 28). Feline oncology horizons have been investigated less so far. To the best of our knowledge, feline mammary gland tumors are the most investigated in comparative feline medicine (29, 30). Ultimate findings in cat oncology showed that mammary gland tumors share a similar basal-like subtype with human breast cancer (29). For instance, Seixas Travassos et al. (30) conducted a retrospective report analyzing the epidemiology, gross morphology, and microscopic features of feline invasive micropapillary carcinoma (IMC), a variant of infiltrating ductal carcinoma of the breast associated with poor outcomes. According to the authors, feline IMC shares similar morphologic and clinical features with women's breast cancer (30).

As widely recognized in the literature, chemical compounds dispersed in the environment contribute, along with genetic factors, to the onset of diseases such as neoplasms (24). To this extent, the Lancet Commission on Pollution and Health estimated that 9 million deaths per year are associated with environmental exposure, including heavy metal pollution (31). In general, metals act by disrupting biological pathways and leading to irreversible biological damage in animals and humans (32–36). Among heavy metals, cadmium has been classified as a human carcinogen capable of inducing melanoma and skin cancer (37). Moreover, metals can accumulate in animal tissues and can be used as potential *biomonitors* (38, 39). Most of the environmental *biomonitors* described in the literature refer to sylvatic animals (40–44). Among all *biomonitors*, wild boars are exposed to heavy metals constantly and can, therefore, accumulate chemicals present in their habitat. In addition, due to the small extension of the area investigated in this study (the Liguria region), wild boars are closely exposed to anthropogenic emissions and share a tight co-existence with human and pet habitats. Thus, biomonitor information may give an *in vivo* perspective of metal accumulation not only in wildlife but also in livestock and human health (45–47).

Overall, the primary focus of the present study is to estimate the most frequent feline cancers occurring in the Liguria region using data collected by the Istituto Zooprofilattico Sperimentale del Piemonte, Liguria, and Valle d'Aosta (IZS PLVA) between 2002 and 2022. First, we aim to evaluate the amount of environmental cadmium pollution extracted from target organs (the liver and the kidney) of wild boars (i.e., *biomonitors*) sampled within Ligurian regional limits. Then, we aim to evaluate the association between the amount of metal concentration in boars and the frequency of tumors in cats. In this context, we sampled cases that shared the same location and coexisted during the same time period (2002–2022).

## 2 Materials and methods

### 2.1 Data collection

A retrospective study was conducted in the Liguria region (northwest Italy) between 2002 and 2022. During this period, the Animal Tumor Registry (ATR) of Genoa collected and analyzed samples of cats with suspected cancer directly from the veterinary clinics of the whole region. The study has focused on cats from Liguria diagnosed with cancer. Diagnoses were classified according to the WHO International Histological Classification of Tumors of Domestic Species (48, 49). Both tumor morphology and topography have been coded using an appropriately adapted ICD-O-3 classification system (50).

To determine the location of the tumor, topographical codes were grouped into 15 groups according to Graf et al. (51) and Grüntzig et al. (52). Tumors of peripheral nerves and autonomic nervous tissues were included together with the soft tissue tumors (51, 52). Furthermore, tumors were categorized according to their anatomic location as external (mammary gland, skin, and male sexual organs) or visceral (bones, joints, cartilage; eye, brain, meninges; endocrine glands; gastrointestinal tract; other female sex organs; respiratory system, intrathoracic organs; retroperitoneum, peritoneum; soft tissues; and urinary organs; see Supplementary Tables 1, 2). The dataset involved a population of 4,399 cats, including 3,195 females (1,425 neutered) and 1,204 males (750 neutered), and individual information about tumor location, ICD-O-3 morphological codes, feline's breed, sex, neuter status, date of birth, date of diagnosis, national territorial unit code of the town of owner's residence, and an alphanumeric string uniquely identifying the owner's surname. Data of all owners were collected, including informed consent for privacy, allowing the use of anonymized protected data for research purposes.

### 2.2 Chemical analysis of metals from *biomonitor* organs

The chemical unit of IZS PLVA extracted cadmium from target organs (i.e., the liver and the kidney) of 185 wild boars, which were passively and actively sampled in the Ligurian territory from 2002 to 2022. Tissue samples were homogenized and then transferred to a Teflon^®^ microwave vessel and mixed with 65% nitric acid (Sigma-Aldrich S.r.l., Milano, cat. V001338) and hydrogen peroxide (Merck Millipore, Germany, cat. 1.086.001.000). The samples were then digested using a laboratory microwave oven. The extract was filtered and diluted with ultrapure water. The determination of Cd^2+^ contents was carried out using the Analytical Yena 650 Plus Atomic Absorption Spectrometer with a graphite furnace at 228.8 nm with a current of 4 mA. The quantification was performed by the standard addition method, adding a certified standard solution purchased from Ultra Scientific to the matrix solution. The data were plotted as absorbance vs. the amount of the standard added. The least squares line intersects the *x*-axis at the negative of the concentration of the sample. The quantification limit (LOQ) was equal to 0.020 mg/kg. To test reagent purity and possible contamination, “blanks” were analyzed at each run using the procedure described as follows.

### 2.3 Statistical analysis

Prior to analytical analyses, the following variables were categorized: tumor site (lymph node; urinary organs; other female sex organs; mammary gland; skin; soft tissue; bones, joints, and cartilage; blood and hemopoietic system; respiratory system; liver and intrahepatic bile ducts; small intestine; gingiva; and others), morphological codes of the ICD-O-3 (epithelial, germ cell, gonadal, lymphoid; melanoma; mesenchymal; neural; odontogenic; and skeletal), age class (0–4 years; 5–8 years; 9–12 years; 13–16 years; and 17–20 years), sex (female; male), neuter status (neutered and not neutered), years of the investigation (2002–2006; 2007–2011; 2012–2016; and 2017–2022). All tumor sites with frequencies <1% have been categorized as “others.” Multiple tumors were discarded and ignored during the analysis. Tumor cases collected from 2002 to 2022 were presented as relative frequencies. Since no cat population was available, we obtained PMRs (Equation 1) by performing Poisson regression over the investigated period (53). The difference between frequencies has been assessed using the Wilcoxon rank-sum test.


(1) $$\begin{array}{l} PMR=\frac{Proportion\;of\;cases\;from\;a\;specific\;tumor\;site}{Proportion\;of\;cases\;from\;all\;tumor\;sites\;recorded} \end{array}$$

Metal information has been obtained from the biomonitors and covers more than 90% of the regional surfaces (Supplementary Table 3). We used a geostatistical interpolation technique known as kriging to estimate missing values. Kriging is a spatial interpolation method that estimates values at unsampled locations based on observed data points within a geographic area. It considers both the spatial correlation between sample points and the spatial variability of the studied phenomenon. In kriging, weights are assigned to nearby sample points based on their distance and spatial correlation with the unsampled location. These weights are optimized to minimize the prediction variance, resulting in a surface that provides the best estimate of the unknown values. Subsequently, cadmium values for Ligurian municipalities were stratified into quartiles and visualized using a heatmap.

To understand how cadmium pollution could contribute to tumor occurrence, we developed a univariate and multivariate mixed-effects Poisson GLM (generalized linear model) to investigate fixed and random effects considering the presence/absence of multiple tumors. Cadmium concentrations, sex, age class, neuter status, and years of the investigation have been identified as *covariates* (*x*_n = 1, …, *k*_), while total cases of tumors have been recorded as an *offset* (E) and *PMR* as an outcome (Equation 2).


(2) $$\begin{array}{l} \frac{e^{\ln\left(E\right)+\beta_{1}x_{1}+\cdots +\beta_{i}\left(x_{i}+1\right)+\cdots +\beta_{k}x_{k}}}{e^{\ln\left(E\right)+\beta_{1}x_{1}+\cdots +\beta_{i}x_{i}+\cdots \beta_{k}x_{k}}}\;=\frac{\;\left(Y|X_{i}+1\right)}{\;\left(Y|X_{i}\right)} \end{array}$$

In sex-specific neoplasms, sex was not considered. Cadmium levels were categorized according to the cadmium meat EU limit, i.e., Regulation (EC) No 853/2004 (54) and Commission Regulation (EC) No 1881/2006 (55) (above and below the EU limit). The level of pollution at the provincial and municipal levels was then evaluated, and the median cadmium concentration for each Ligurian province and municipality was calculated. All statistical analyses were carried out using STATA 17.0 (Stata Corp., Texas, USA) and R Studio^®^.

## 3 Results

### 3.1 Descriptive analysis

**Figure 1:**
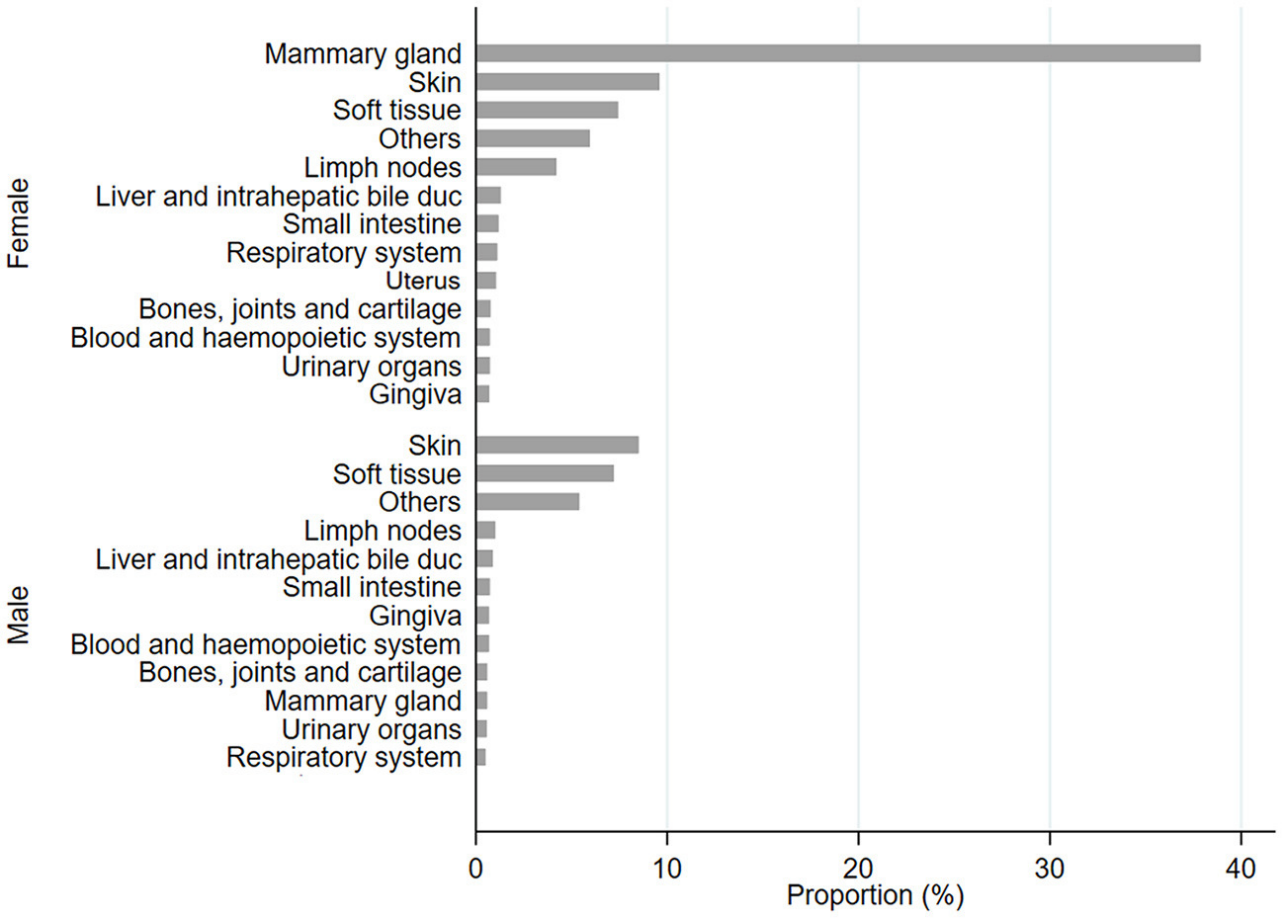
Proportion, expressed as a percentage, of diagnosis of tumor sites in female (F) and male (M) populations.

From 2002 to 2022, the NILOV database collected 4,399 diagnoses within the Ligurian territories; most were females (72.63%), and half the population was neutered (49.44%). In Figure 1, all tumoral proportions were expressed as percentages.

**Figure 2:**
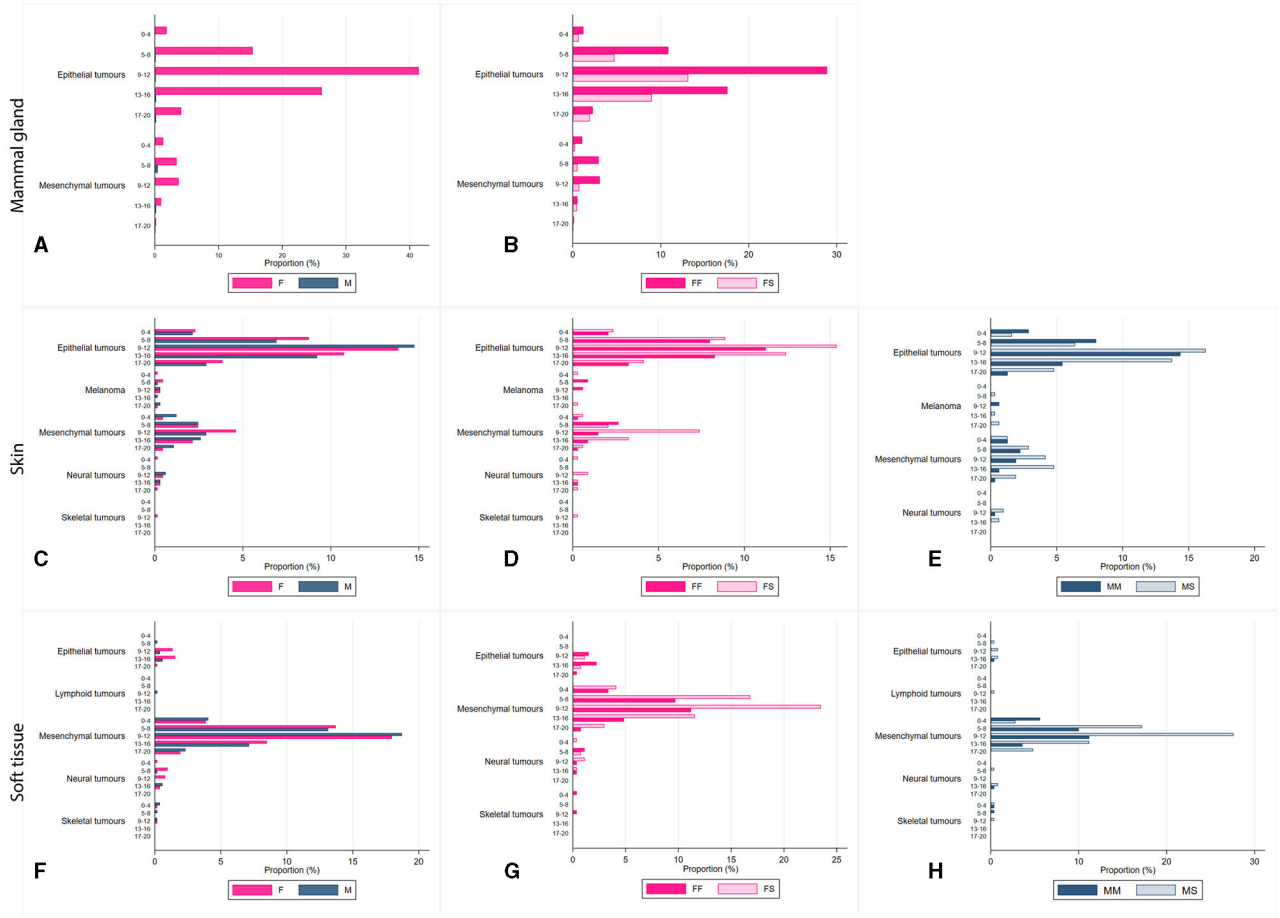
Proportion, expressed as a percentage, of diagnosis of tumors located in the mammary glands **(A, B)**, the skin **(C–E)**, soft tissue **(F–H)**, by age class (0-4 years old; 5-8 years old; 9-12 years old; 13-16 years old; and 17–20 years old), and sex [**(B, D, G)** FF, entire female; FS, sterilized female; **(E, H)** MM, entire male; MS, sterilized male].

In female cats, the most frequent sites affected by tumors were the mammary gland, skin, and soft tissue; meanwhile, for male cats, the frequently affected sites were skin and soft tissue. All data have been stratified by age class, and it was observed that in all cases, frequencies were significantly higher for 9–12-year-old cats (*p* < 0.0001; Figure 2 and Supplementary Table 4). Cases have been stratified by sex (Figures 2A, C, F) and neutering status (Figures 2B, D, E, G, H). As shown in Figure 2A, tumors in mammary glands occur mainly in female cats compared to males, as confirmed using the chi-squared test (*p* < 0.0001; Figure 2B). Focusing on the neutering status, it has been possible to observe that the proportion is higher in not neutered cats. Meanwhile, cases of tumors in the skin (Figures 2D, E) and soft tissue (Figures 2G, H) sites are more commonly observed in neutered than in the not-neutered ones, both in males and females. These results were confirmed by the Wilcoxon rank-sum test, as *p*-values resulted in <0.0001 (Supplementary Table 5). The tumor's localization has been studied for the three most frequent tumors, as presented in Figure 2.

Two main tumor sites were observed within mammary gland tumors: epithelial and mesenchymal, as shown in Figures 2A, B. In contrast, soft tissue cancers displayed heterogeneous localization, including epithelial, mesenchymal, neural, skeletal, and lymphoid. Skin tumors were found mainly in epithelial, mesenchymal, melanoma, neural, and skeletal sites (Figures 2C–E).

### 3.2 Association between cadmium pollution and neoplasia occurrence

**Figure 3:**
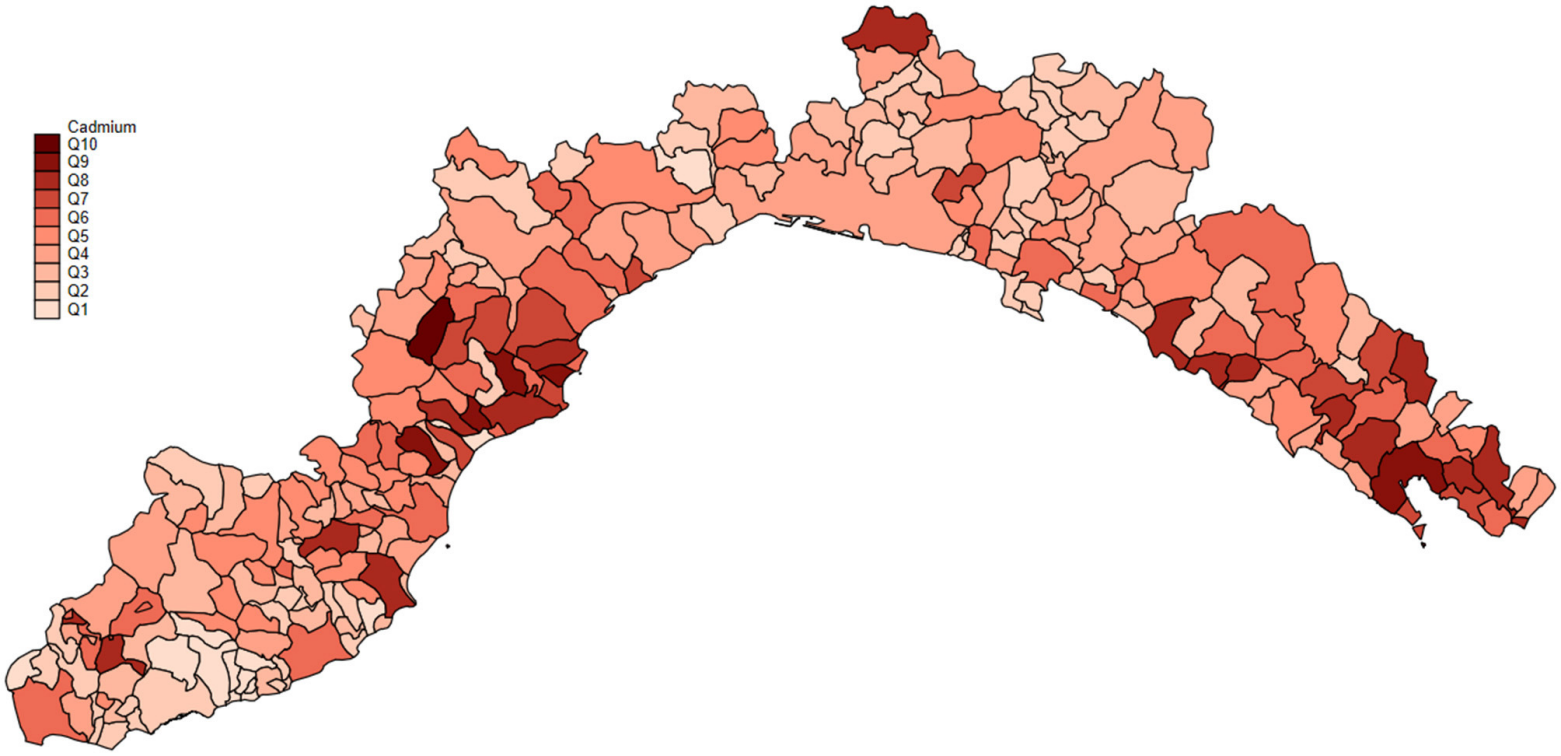
Cadmium concentration of all the Ligurian municipalities, represented in quartiles Q1–10. Q1 = 6.76; Q2 = 2.6–3.63; Q3 = 1.87–2.42; Q4 = 1.39–1.7; Q5 = 1.06–1.36; Q6 = 0.82–1.05; Q7 = 0.62–0.79; Q8 = 0.44–0.61; Q9 = 0.27–0.42; and Q10 = 0.002–0.250.

We effectively estimated missing values across the regional surfaces, ensuring comprehensive coverage of the analysis by leveraging the spatial information available from the *biomonitors'* data. Among all the municipalities investigated for heavy metal presence, cadmium concentrations were above the EU limit (56) for 72 municipalities and below for 113 municipalities. The mean cadmium concentration, collected from biomonitors during 2002–2022, was 0.53 ± 0.40 mg/kg. The mean cadmium concentration in the Genova province was 0.99 mg/kg (95% CI: from 0.10 to 0.94), in Imperia was 0.99 mg/kg (95% CI: from 0.11 to 0.90), in La Spezia was 0.93 mg/kg (95% CI: from 0.10 to 0.86) and in Savona was 0.89 mg/kg (95% CI: from 0.93 to 0.85). Overall, the data presented here suggest that municipalities within La Spezia and Savona provinces represent the Ligurian areas with higher cadmium concentrations (Figure 3).

**Figure 4:**
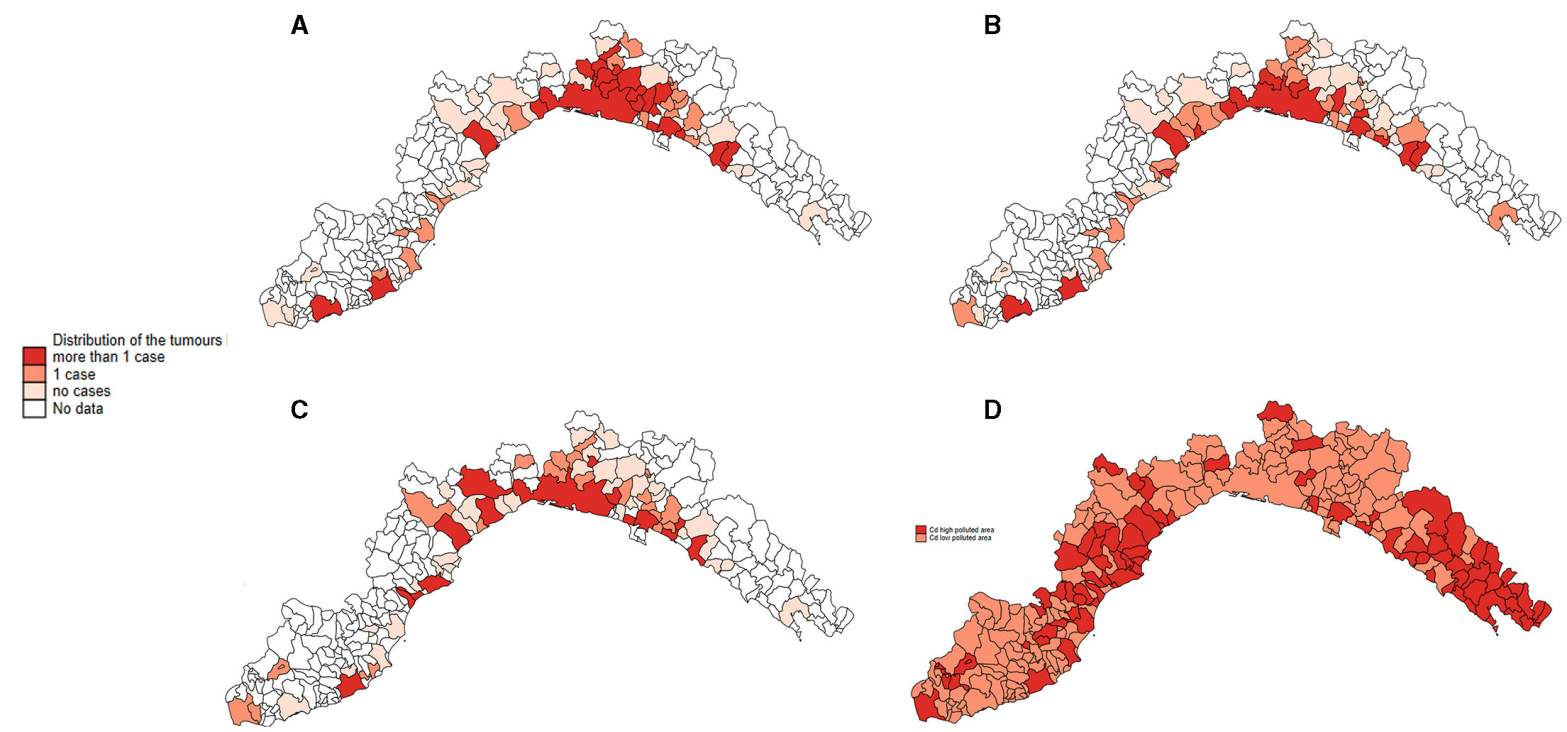
Distribution of all the Ligurian municipalities, represented as the relative frequency of tumors located in the mammary gland **(A)**, the skin **(B)**, soft tissue **(C)**, and the heat map of the 2-level of cadmium pollution **(D)**.

In all Poisson GLM models, tumors had significantly higher proportional morbidity ratios (PMRs) in higher cadmium-polluted areas compared to lower cadmium-polluted areas (Table 1). The age class variable did not show significant PMRs in all models. Sex covariate had significant PMRs: females had PMRs lower than males for tumors in soft tissue and skin sites (in multivariate models: 0.60, 95% CI: 0.48–0.74 and 0.65, 95% CI: 0.54–0.79, respectively). Neuter status was a significant variable, except for skin tumors. Neutered cats had lower PMRs than not neutered ones in the mammary gland tumors model (0.58, 95% CI: 0.47–0.72 in the univariate model and 0.53, 95% CI: 0.43–0.44 in the multivariate model). In soft tissue tumors, neutered cats showed higher PMRs (1.56, 95% CI:1.15–2.11 in the univariate model and 2.26, 95% CI: 1.86–2.73 in the multivariate model). The covariate of the years of the investigation had no significant PMRs for tumors in the mammary gland and skin. All tumor frequencies are reported in Figure 4 as a heatmap.

**Table 1:** The results of the Poisson models, including cadmium concentration, age class, sex, neuter status, and investigated year.

**Variable**	**PMRs proportional morbility ratios (95% CI)**					
	**Mammary gland tumors response**		**Soft tissue tumors response**		**Skin tumors response**	
	**Univariate**	**Multivariate**	**Univariate**	**Multivariate**	**Univariate**	**Multivariate**
Cd low polluted area	Ref.	Ref.	Ref.	Ref.	Ref.	Ref.
Cd high polluted area	42.61 (29.94–60.65)	44.94 (31.45–64.22)	66.81 (48.80–91.47)	56.28 (40.94–77.35)	78.34 (60.14–102.05)	80.13 (61.09–105.11)
0–4 years old	Ref.	Ref.	Ref.	Ref.	Ref.	Ref.
5–8 years old	1.08 (0.60–1.96)	1.00 (0.56–1.78)	1.62 (0.97–2.69)	1.46 (0.87–2.43)	1.16 (0.72–1.88)	1.21 (0.76–1.91)
9–12 years old	1.41 (0.82–2.41)	1.35 (0.79–2.30)	1.18 (0.72–1.94)	1.10 (0.67–1.81)	0.99 (0.63–1.55)	1.06 (0.69–1.64)
13–16 years old	1.28 (0.74–2.20)	1.25 (0.73–2.14)	0.85 (0.51–1.41)	0.85 (0.51–1.41)	0.91 (0.57–1.43)	0.95 (0.61–1.48)
17–20 years old	1.20 (0.66–2.18)	1.18 (0.66–2.14)	0.43 (0.22–0.86)	0.53 (0.27–1.05)	1.13 (0.68–1.88)	1.18 (0.72–1.92)
Male	–	–	Ref.	Ref.	Ref.	Ref.
Female	–	–	0.57 (0.46–0.70)	0.60 (0.48–0.74)	0.63 (0.51–0.76)	0.65 (0.54–0.79)
Not neutered	Ref.	Ref.	Ref.	Ref.	Ref.	Ref.
Neutered	0.58 (0.47–0.72)	0.53 (0.43–0.64)	1.56 (1.15–2.11)	2.26 (1.86–2.73)	1.25 (0.97–1.63)	1.16 (0.89–1.51)
2002–2006	Ref.	Ref.	Ref.	Ref.	Ref.	Ref.
2007–2011	0.87 (0.64–1.18)	0.93 (0.69–1.26)	0.46 (0.31–0.68)	0.61 (0.41–0.91)	0.87 (0.63–1.20)	1.11 (0.81–153)
2012–2016	0.80 (0.59–1.07)	0.83 (0.61–1.11)	0.34 (0.22–0.52)	0.46 (0.29–0.71)	0.97 (0.73–1.31)	1.22 (0.91–1.63)
2017–2022	0.90 (0.58–1.41)	0.86 (0.55–1.33)	0.22 (0.10–0.47)	0.24 (0.11–0.51)	2.00 (1.46–2.75)	1.97 (1.47–2.64)

## 4 Discussion

Cats are useful sentinels for human health and environmental exposure to toxic and cancerogenic chemicals (12, 29, 57–59). Within a critical One Health approach, pets share indoor and outdoor environmental risks with their owners, providing translational evidence of possible positive implications for human health. In current times, pet translation medicine is becoming an important medical branch, and the pet tumor registry offers a rich prevention overview for clinical veterinaries (2, 29, 57–61). In our dataset, female cats are the most represented, accounting for 72.6% of the total recorded tumor cases. These results are supported by previous findings, where female tumors account for 51.8%−62.3% of all tumors (59). In our dataset, the mammary gland, soft tissue, and skin cancers are the most frequent, in accordance with previously published literature (58, 59, 62, 63). In two Italian studies, the prevalence of mammary gland tumors was estimated to be 11%−16.3%, and the prevalence of skin and soft tissue was estimated to be 55.1% (17, 59). In veterinary comparative oncology, feline mammary gland tumors are the most investigated tumors and primarily affect females (12, 29, 57, 59, 60). Conversely, the risk of tumors in soft tissue and skin is lower in the female population. In addition, the results suggest the importance of ovariohysterectomy in reducing the risk of developing mammary gland tumors in female cats, as documented by previous studies (57, 60). Our findings suggest that neuter status plays a crucial role in the development of mammary gland tumors. According to our results, entire females have significantly higher PMRs (PMR neutered vs. not neutered = 0.58, 95% CI: 0.47–0.72 in the univariate model and 0.53, 95% CI:0.43–0.64 in the multivariate model) compared to neutered ones, suggesting that hormonal influences are likely involved in the pathogenesis of mammary gland tumors (60). The key role of neutering was already hypothesized by Overley et al.: ovariohysterectomy within 6 months or 1 year of life reduces the risk of developing mammary gland tumors by ~91%−86% (57). On the contrary, our findings suggest that sterilization could be a risk factor for the development of skin and soft tissue cancers (Table 1). To the best of our knowledge, the effect of neutering in these two body sites has been studied little and suggests the involvement of sexual hormones in the development of tumors (64, 65). Further detailed *in vitro* and *in vivo* studies are needed to support and understand the biological processes involved (66).

The inclusion of the diagnostic year in the models reported no significant values, except for soft tissue tumors, which could be because no significant improvements have been made to the diagnostic techniques. From 2002 to 2022, all tumors were diagnosed by staining samples with the hematoxylin-eosin technique. This result is in accordance with Graf et al.'s study (51).

An investigation of cadmium pollution and cancer cases highlighted a higher proportion of tumor cases in the most polluted area (67–71). In this study, environmental information was obtained by wildlife biomonitors sampled within the Ligurian territories. Liguria is a small Italian region (only 5,418 km^2^), where the proximity of rural areas and cities facilitates a miscellany of pets and sylvatic animals. Wild boars, as consolidated in scientific literature, are good environmental *biomonitors* that are useful for monitoring persistent pollutants in the habitat where ungulates live (40, 40–42, 72, 73).

Cats and wild boars coexisted during the investigated period (2002–2022) and shared in both the rural and urban areas. In particular, many wild boar groups inhabit both the rural and urban zones; for example, in Genoa, wild boar populations permanently reside along riverbeds. Both of these species are susceptible to metal pollution, as reported in the literature (32, 74–77); this is because natural and anthropogenic sources release cadmium in the atmosphere, which can be transported through air particulates and soil. Metals, which are mainly released through human activities, accumulate in the target organs (i.e., the liver and the kidney) throughout physiological bioaccumulation (40, 42, 73). Indeed, biological mechanisms in wildlife animals do not allow cadmium disposal, and it bioaccumulates in tissues (73). In our study, by comparing tumor relative proportion with cadmium polluted level, we observed that cats that live in higher polluted areas (cadmium concentration higher than 0.50 mg/kg) have significantly higher PMRs of developing tumors compared to those living in less polluted areas (Table 1). Interestingly, similar findings regarding humans have already been reported in the scientific literature (67–71). Recently, García-Pérez et al. (67) reported a relative risk of 1.12 (95%CI: from 1.00 to 1.26) in areas near cadmium sources, and McElroy et al. (68) found that higher cadmium intake correlated with increased breast cancer risk. Animal studies have shown that acute cadmium exposure increases the density of epithelial cells in breast cancer progression (71).

The association between cadmium exposition and cancer occurrence is a debated topic, and the relevance of acute vs. chronic exposure is a worthwhile question (71). As noted by many authors, most of the latest environmental studies analyze the carcinogenic potential of cadmium, focusing exclusively on acute metal exposures, and few studies have investigated the effects of chronic low-level cadmium exposure on breast cancer development (71, 78). *In vivo* and *in vitro* experiments highlighted the importance of chronic exposure studies (71, 78, 79). Ponce and colleagues (71), in a case-control study, experimented that both acute and chronic exposure downregulates gene expression, affecting breast cancer cells. Franzoni et al. (79) observed that chronic Cd^2+^ exposure leads to an immunosuppressive status and increases infection susceptibility. Similarly, Tamás et al. (35) proved that a trigger degenerative disease is caused by chronic Cd^2+^ exposure, which is associated with protein misfolding. Our results obtained from feline cases support scientific evidence of detrimental damages caused by long-term chronic exposition, in accordance with previously published literature reporting a biological half-life of up to 30 years (67, 71, 80, 81). In our NILOV data, most of the cancer onsets appear approximately in 9–12 years old, as shown in Figure 2, and as already reported for dogs by Crescio et al. (22). This outcome, supported by recent studies (35, 36, 82), suggests that prolonged exposure of felines to cadmium pollution for at least 9 years may cause highly sensitive receptors to metals, resulting in a gradual deterioration of health and, in the end, leading to the development of neoplasia.

## 5 Conclusion

Within a critical One Health approach, cats share indoor and outdoor environmental risks with humans, and feline cancer studies may give positive insights into human health. Regarding cat tumors, data shows that tumors of epithelial and mesenchymal locations are the most frequent among skin and soft tissue tumors within the feline population (57–60, 62, 63). Metal pollution data highlight that the provinces of La Spezia and Savona are the most polluted in Liguria, and the municipalities with the highest tumor frequency are the same as those with the highest cadmium levels. Ovariohysterectomized females have a lower probability of developing mammary gland cancer than entire ones, and this finding is consistent with recent literature (52). On the other hand, neutered cats are more likely to develop soft tissue and skin cancers, but additional investigation is needed to understand the biological mechanisms involved. However, the present study presents several limitations. First, the lack of cat population across the investigated area prevents further investigation. Second, limited knowledge about the kinetics of the absorption of biological tissue limits assumptions about the quantification of metal exposure.

Government authorities, public and private diagnostic laboratories, and scientific institutes should collaborate to obtain a complete Italian ATR to better study pets as human health sentinels. Pet epidemiology could play a considerable role in translational medicine. Nonetheless, to assess the nationwide incidence of tumors, it is necessary to optimize and harmonize data collection (83).

## Data availability statement

The original contributions presented in the study are included in the article/Supplementary material, further inquiries can be directed to the corresponding author.

## Ethics statement

Ethical approval was not required for the study involving animals in accordance with the local legislation and institutional requirements because the Ethics Committee considered, on 03/03/2023, (protocol number: 0003275) that it was not necessary to resort to specific evaluation by a body responsible for animal welfare.

## Author contributions

RG: Conceptualization, Data curation, Formal analysis, Investigation, Methodology, Writing – original draft, Writing – review & editing. FD: Conceptualization, Data curation, Formal analysis, Investigation, Methodology, Writing – review & editing. LD: Supervision, Validation, Writing – review & editing. MC: Supervision, Validation, Writing – review & editing. VC: Writing – review & editing. BV: Writing – review & editing. ER: Writing – review & editing, Funding acquisition, Project administration.
