# Supplementary material for: Mammary gland, skin and soft tissue tumors in pet cats: findings of the feline tumors collected from 2002 to 2022
Source: Front Vet Sci. 2024 Aug 14;11:1320696. doi: 10.3389/fvets.2024.1320696 (PMC11349711; doi:10.3389/fvets.2024.1320696)
Supplement: Supplementary file 1 [file Data_Sheet_1.docx]

Supplementary Material

Supplementary Table 1: Coding of tumour locations according to Grüntzig et al. (52)

| **Location** | **ICD-9** | **ICD-10** |
| --- | --- | --- |
| Blood, haemopoietic system | T 169.0 - 169.9 | C 42 |
| Neoplasia of bones, joints, cartilage | T 170.0 - 170.9 | C 40-41 |
| Brain, meninges, other parts of CNS | T 190.0 - 192.9 | C 70-72 |
| Mammary gland | T 174.0 - 175.9 | C 50 |
| Endocrine gland | T 193.0 - 194.9 | C 73-75 |
| Gastrointestinal tract | T 150.0 - 159.9 (158 excluded) | C 16-26.8 |
| Lymph nodes | T 196.0 - 196.9 | C 77 |
| Male sexual organs | T 185.0 - 186.9 ; T 187.1 - 187.9 | C 60-63.2 |
| Oral cavity, pharynx | T 140.0 - 149.9 | C 2.9-11 |
| Other female sex organs | T 179.0 -184.9 | C 51-58 |
| Respiratory system, intrathoracic organs | T 160.0 - 165.9 | C 30-39 |
| Retroperitoneum, peritoneum | T 158 | C 48 |
| Skin | T 173.0 - 173.9 | C 44 |
| Soft tissues | T 171.0 - 171.9 | C 49; C47 |
| Urinary organs | T 188.0 - 189.9 | C 67-68 |

Supplementary Table 2: Variables considered during the descriptive analysis of the diagnosticated cancers collected by the NILOV between 2002 and 2022 (n = 4399).

| **Characteristics** | **Number** | **Percentage (%)** |
| --- | --- | --- |
| **Tumour site** | | |
| Lymph nodes | 230 | 5.23 |
| Urinary organs | 57 | 1.30 |
| Other female sex organs | 46 | 1.05 |
| Mammary gland | 1692 | 38.46 |
| Skin | 796 | 18.10 |
| Soft tissue | 644 | 14.64 |
| Bones, joints and cartilage | 60 | 1.36 |
| Blood and haemopoietic system | 62 | 1.41 |
| Respiratory system | 71 | 1.61 |
| Liver and intrahepatic bile ducts | 96 | 2.18 |
| Small intestine | 84 | 1.91 |
| Gingiva | 61 | 1.39 |
| Others | 500 | 11.37 |
| **Tumour origin** | | |
| Epithelial | 2198 | 49.97 |
| Germ cell | 5 | 0.11 |
| Gonadal | 10 | 0.23 |
| Lymphoid | 22 | 0.50 |
| Melanoma | 33 | 0.75 |
| Mesenchymal | 929 | 21.12 |
| Neural | 29 | 0.76 |
| Odontogenic | 2 | 0.05 |
| Skeletal | 46 | 1.05 |
| Not defined | 1125 | 25.57 |
| **Spayed status** | | |
| Spayed | 2175 | 49.44 |
| Not spayed | 2224 | 50.56 |
| **Gender** | | |
| Female | **3195** | **72.63** |
| Male | 1204 | 27.37 |
| **Age class**  **(years)** | | |
| 0-4 | 306 | 6.96 |
| 5-8 | 901 | 20.48 |
| 9-12 | 1753 | 39.85 |
| 13-16 | 1146 | 26.05 |
| 17-20 | 293 | 6.66 |

Supplementary Table 3: Cadmium concentration expressed as average of all wild boars sampled within Ligurian municipalities (n/a: data not available).

| **Municipalities** | **Cd (mg/kg)** | **Municipalities** | **Cd (mg/kg)** |
| --- | --- | --- | --- |
| Airole | n/a | Masone | 0.54 |
| Alassio | 0.33 | Massimino | 0.28 |
| Albenga | 0.65 | Mele | 0.24 |
| Albisola Superiore | 0.62 | Mendatica | 0.23 |
| Albissola Marina | n/a | Mezzanego | 0.36 |
| Altare | 0.32 | Mignanego | 0.24 |
| Ameglia | 0.64 | Millesimo | 0.44 |
| Andora | 1.05 | Mioglia | 0.2 |
| Apricale | 1.3 | Moconesi | 0.3 |
| Aquila d'Arroscia | 0.51 | Molini di Triora | 0.24 |
| Arcola | 1.12 | Moneglia | 1.08 |
| Arenzano | 0.15 | Montalto Ligure | 0.49 |
| Armo | n/a | Montebruno | 0.15 |
| Arnasco | 0.26 | Montegrosso Pian Latte | 0.44 |
| Aurigo | 0.43 | Monterosso al Mare | n/a |
| Avegno | n/a | Montoggio | 0.28 |
| Badalucco | 0 | Murialdo | 0.36 |
| Bajardo | 0.28 | Nasino | 0.53 |
| Balestrino | 0.54 | Ne | 0.43 |
| Bardineto | 0.47 | Neirone | 0.21 |
| Bargagli | 0.46 | Noli | 0.94 |
| Bergeggi | 0.57 | Olivetta San Michele | 0.05 |
| Beverino | 0.6 | Onzo | 0.32 |
| Bogliasco | n/a | Orco Feglino | 1.74 |
| Boissano | 0.27 | Orero | 0.31 |
| Bolano | n/a | Ortonovo | n/a |
| Bonassola | n/a | Ortovero | 0.55 |
| Bordighera | n/a | Osiglia | 3.38 |
| Borghetto d'Arroscia | 0.49 | Ospedaletti | 0.2 |
| Borghetto di Vara | 0.97 | Pallare | 0.63 |
| Borghetto Santo Spirito | n/a | Perinaldo | 0.3 |
| Borgio Verezzi | 0.68 | Piana Crixia | 0.5 |
| Borgomaro | 0.29 | Pietra Ligure | 0.06 |
| Bormida | 1.01 | Pietrabruna | n/a |
| Borzonasca | 0.22 | Pieve di Teco | 0.47 |
| Brugnato | 0.15 | Pieve Ligure | n/a |
| Busalla | 0.16 | Pigna | 0.37 |
| Cairo Montenotte | 0.39 | Pignone | 1.16 |
| Calice al Cornoviglio | 1.32 | Plodio | 0.41 |
| Calice Ligure | 0.14 | Pompeiana | n/a |
| Calizzano | 0.53 | Pontedassio | 0.23 |
| Camogli | n/a | Pontinvrea | 0.58 |
| Campo Ligure | 0.42 | Pornassio | 0.22 |
| Campomorone | 0.26 | Portofino | n/a |
| Camporosso | 0.38 | Portovenere | 0.84 |
| Carasco | 0.61 | Prelà | 0.42 |
| Caravonica | 0.76 | Propata | n/a |
| Carcare | 0.23 | Quiliano | 0.85 |
| Carpasio | 0.49 | Ranzo | 0.29 |
| Carro | 0.73 | Rapallo | 0.58 |
| Carrodano | 0.67 | Recco | 0.32 |
| Casanova Lerrone | 1.05 | Rezzo | 0.49 |
| Casarza Ligure | 0.3 | Rezzoaglio | 0.37 |
| Casella | 0.15 | Rialto | 0.6 |
| Castel Vittorio | 0.6 | Riccò del Golfo di Spezia | 1.31 |
| Castelbianco | 0.48 | Riomaggiore | n/a |
| Castellaro | n/a | Riva Ligure | n/a |
| Castelnuovo Magra | n/a | Roccavignale | 0.33 |
| Castelvecchio di Rocca Barbena | 0.59 | Rocchetta di Vara | 0.83 |
| Castiglione Chiavarese | 0.72 | Rocchetta Nervina | n/a |
| Celle Ligure | 1.01 | Ronco Scrivia | 0.33 |
| Cengio | 0.25 | Rondanina | n/a |
| Ceranesi | 0.34 | Rossiglione | 0.25 |
| Ceriale | 0.36 | Rovegno | 0.26 |
| Ceriana | n/a | San Bartolomeo al Mare | 0.1 |
| Cervo | 0.23 | San Biagio della Cima | n/a |
| Cesio | n/a | San Colombano Certenoli | 0.33 |
| Chiavari | 0.63 | San Lorenzo al Mare | n/a |
| Chiusanico | 0.24 | Sanremo | 0.17 |
| Chiusavecchia | n/a | Santa Margherita Ligure | n/a |
| Cicagna | 0.42 | Santo Stefano al Mare | n/a |
| Cipressa | 0.3 | Santo Stefano d'Aveto | 0.33 |
| Cisano sul Neva | 0.54 | Santo Stefano di Magra | 0.52 |
| Civezza | n/a | Sant'Olcese | 0.3 |
| Cogoleto | 0.38 | Sarzana | 1.34 |
| Cogorno | 0.34 | Sassello | 0.45 |
| Coreglia Ligure | 0.28 | Savignone | 0.25 |
| Cosio d'Arroscia | n/a | Savona | 0.58 |
| Cosseria | n/a | Seborga | n/a |
| Costarainera | n/a | Serra Riccò | 0.18 |
| Crocefieschi | n/a | Sesta Godano | 0.5 |
| Davagna | 0.84 | Sestri Levante | 1.12 |
| Dego | 0.15 | Soldano | 0.44 |
| Deiva Marina | 1.09 | Sori | 0.63 |
| Diano Arentino | n/a | Spotorno | 1.74 |
| Diano Castello | 0.25 | Stella | 0.36 |
| Diano Marina | n/a | Stellanello | 0.42 |
| Diano San Pietro | n/a | Taggia | n/a |
| Dolceacqua | n/a | Terzorio | n/a |
| Dolcedo | 0.31 | Testico | 0.55 |
| Erli | 0.58 | Tiglieto | 0.19 |
| Fascia | n/a | Toirano | 1.55 |
| Favale di Malvaro | 0.48 | Torriglia | 0.43 |
| Finale Ligure | 1.11 | Tovo San Giacomo | 1.82 |
| Follo | n/a | Tribogna | n/a |
| Fontanigorda | 0.21 | Triora | 0.37 |
| Framura | 0.39 | Urbe | 0.11 |
| Garlenda | 0.3 | Uscio | 0.22 |
| Genova | 0.34 | Vado Ligure | 1.21 |
| Giustenice | 1.06 | Valbrevenna | 0.5 |
| Giusvalla | 0.57 | Vallebona | 0.26 |
| Gorreto | 0.15 | Vallecrosia | n/a |
| Imperia | 0.59 | Varazze | 0.35 |
| Isola del Cantone | 1.14 | Varese Ligure | 0.58 |
| Isolabona | 0.62 | Vasia | n/a |
| La Spezia | 1.53 | Vendone | 0.37 |
| Laigueglia | 0.24 | Ventimiglia | 0.62 |
| Lavagna | n/a | Vernazza | n/a |
| Leivi | 0.16 | Vessalico | 0.33 |
| Lerici | 0.97 | Vezzano Ligure | 0.7 |
| Levanto | 0.44 | Vezzi Portio | 0.73 |
| Loano | 0.99 | Villa Faraldi | 0.46 |
| Lorsica | 0.26 | Villanova d'Albenga | 0.41 |
| Lucinasco | n/a | Vobbia | 0.34 |
| Lumarzo | 0.37 | Zignago | 0.29 |
| Magliolo | 0.46 | Zoagli | 0.25 |
| Maissana | 0.24 | Zuccarello | 0.45 |
| Mallare | 0.79 |  |  |

Supplementary Table 4: Chi-squared test of the tumor cases over age class.

| **Anatomical location** | **Affected** | **Age class** | | | | | **Total** | **Chi-squared test (p-value)** |
| --- | --- | --- | --- | --- | --- | --- | --- | --- |
|  |  | 0 - 4 | 5 - 8 | 9 - 12 | 13 - 16 | 17- 20 |  |  |
| **Mammary gland** | **Not** | 224 | 546 | 913 | 631 | 176 | 2490 | <0.001 |
|  | **Yes** | 53 | 293 | 719 | 449 | 90 | 1604 |  |
| **Skin** | **Not** | 222 | 684 | 1369 | 892 | 194 | 3361 | <0.001 |
|  | **Yes** | 55 | 155 | 263 | 188 | 72 | 733 |  |
| **Soft tissue** | **Not** | 223 | 667 | 1393 | 958 | 242 | 3483 | <0.001 |
|  | **Yes** | 54 | 172 | 239 | 122 | 24 | 611 |  |

Supplementary Table 5: Wilcoxon rank-sum test output results.

|  | **Sex** | **Obs** | **Rank sum** | **Expected rank sum** | **neutered status** | **Obs** | **Rank sum** | **Expected rank sum** | **Wilcoxon rank-sum test (p-value)** |
| --- | --- | --- | --- | --- | --- | --- | --- | --- | --- |
| **Mammary gland** | F | 2973 | 6937132 | 6087217 | **not neutered** | 2064 | 4740532 | 4226040 | <0.001 |
|  | M | 1121 | 1445333 | 2295248 | **neutered** | 2030 | 3641933 | 4156425 |  |
|  | **Combined** | **4094** | **8382465** | **8382465** | **Combined** | **4094** | **8382465** | **8382465** |  |
| **Skin** | F | 2973 | 5785708 | 6087218 | **not neutered** | 2064 | 4032509 | 4226040 | <0.001 |
|  | M | 1121 | 2596757 | 2295247 | **neutered** | 2030 | 4349956 | 4156425 |  |
|  | **Combined** | **4094** | **8382465** | **8382465** | **Combined** | **4094** | **8382465** | **8382465** |  |
| **Soft tissue** | F | 2973 | 5805348 | 6087218 | **not neutered** | 2064 | 3998747 | 4226040 | <0.001 |
|  | M | 1121 | 2577117 | 2295247 | **neutered** | 2030 | 4383718 | 4156425 |  |
|  | **Combined** | **4094** | **8382465** | **8382465** | **Combined** | **4094** | **8382465** | **8382465** |  |
